# AMMI an GGE biplot analysis of grain yield for drought-tolerant maize hybrid selection in Inner Mongolia

**DOI:** 10.1038/s41598-023-46167-z

**Published:** 2023-11-01

**Authors:** Yipu Li, Haizhu Bao, Zhenghan Xu, Shuping Hu, Jiying Sun, Zhigang Wang, Xiaofang Yu, Julin Gao

**Affiliations:** 1 Agricultural College, Inner Mongolia Agricultural University, Hohhot, Inner Mongolia China; 2 Region Research Center for Conservation and Utilization of Crop Germplasm Resources in Cold and Arid Areas, Hohhot, Inner Mongolia China; 3 Vocational and Technical College, Inner Mongolia Agricultural University, Baotou, Inner Mongolia China

**Keywords:** Agricultural genetics, Plant breeding

## Abstract

Due to the ongoing global warming, maize production worldwide is expected to be heavily inflicted by droughts. The grain yield of maize hybrids is an important factor in evaluating their suitability and stability. In this study, we utilized the AMMI model and GGE biplot to analyze grain yield of 20 hybrids from the three tested environments in Inner Mongolia in 2018 and 2019, aiming at selecting drought-tolerant maize hybrids. AMMI variance analysis revealed highly significant difference on main effects for genotype, environment, and their interaction. Furthermore, G11 (DK159) and G15 (JKY3308) exhibited favorable productivity and stability across all three test environments. Moreover, G10 (LH1) emerged as the most stable hybrid according to the AMMI analysis and the GGE biplot. Bayannur demonstrated the highest identification ability among the three tested sites. Our study provides accurate identification for drought-resilient maize hybrids in different rain-fed regions. These findings can contribute to the selection of appropriate hybrids that exhibit productivity, stability, and adaptability in drought-prone conditions.

## Introduction

Water scarcity, large fluctuations in weather patterns, and the unpredictable nature of drought pose a crucial threat to maize production worldwide^1,2^. Maize is the most important cereal crop in China, with the latest estimation indicating a total annual production of approximately 270–280 million tons^3^. In 2021, there were 175 million spring maize among them. Drought-related production losses range from 5 to 30% annually in China's northwest and southwest maize-producing regions, which provide almost one-third of the country's spring maize grain yield^4^. Maize is such a substantial crop for both food and feed purposes in the world, there is tremendous interest in and demand for improving maize drought tolerance^5^.

In the multi-location trials of crop breeding, evaluating yield potential and stability is crucial for assessing the value of promoting new varieties^6,7^. Variance analysis and multiple comparisons of yield allow for easy measurement of yield differences among varieties. However, the stability of varieties primarily arises from the genotype-environment interaction (G × E) effect. G × E analysis enable to evaluate genotype stability and adaptability in terms of yield and yield-related traits^8,9^. Genotype effects are influenced by both genotype and environment, as well as their interaction^10^. The environment often impacts the potential genetic effects of traits, particularly in interfering with artificial selection of quantitative traits such as yield, leading to diminished genetic effects in the offspring^7^. G × E analysis aids breeders in evaluating new varieties in representative growing environments, facilitating the identification of varieties with broad adaptability^11,12^. Moreover, G × E analysis also holds significant value in the final selection stage of core breeding materials.

Currently, there are several statistical methods widely used to analyze G × E effects, including the additive main effects and multiplicative interaction model (AMMI) and the genotype + genotype-by-environment (GGE) biplot analysis^13,14^. The AMMI analysis utilizes principal component analysis (PCA) to minimize the dimensionality of the data. This model is extensively applied in analyzing yield traits across multi-location in variety selecting trials and breeding test experiments. On the other hand, the GGE biplot provides a graphical representation of G × E effects, allowing for visual identification of patterns, relationships, and outliers, facilitating the interpretation and communication of results. However, both AMMI^15^ and GGE^16,17^ model are only representative when two principal components (PCs) are significant. To address this, optimizations have been made, such as the use of two PCs for AMMI stability index (ASI) and AMMI stability value (ASV), in order to better showcase genotype stability^18,19^. Furthermore, several selection indices have been developed to choose genotypes with high yield stability, such as Bajpai Index^20^, simultaneous selection index (SSI)^21^, and non-parametric genotype selection index^22^, which utilize stability parameters and grain yield data to guide simultaneous selection for stability and high yield^23^.

Multi-location trials for selecting drought tolerant maize hybrid were conducted in Inner Mongolia during the year 2018 and 2019. In this study, the AMMI model and GGE biplot were applied to assess grain yield data obtained from 3 rain-fed environments over two years trails. The main objectives were to evaluate the impact of G × E interaction and identify drought tolerant genotypes with high grain yield, stability and narrow or broad adaptability. Additionally, the study aimed to provide drought-resilient hybrids for spring maize cultivation in rain-fed regions of China or similar environments.

## Materials and methods

### Plant materials and experimental site

A total of 20 hybrids, including 19 tested hybrids and one control hybrid (XY335), were used in the drought-tolerant maize hybrid selection trials conducted in Inner Mongolia. The experiment took place over two planting seasons in 2018 and 2019. The hybrids were planted at three different locations: Hangjinhouqi Experimental Station of Bayannur Academy of Agriculture and Animal Husbandry Sciences, Bayannur (107.15°, 40.88°, sandy soil with a pH of 7.4, bulk density of 1,584 kg.m^−3^, organic carbon content of 0.17% m/m, and organic matter content of 13.15 g/kg); Maize Research Center of Inner Mongolia Agricultural University, Salaqi (110.52°, 40.56°, sandy soil with a pH of 7.2, bulk density of 1,578 kg.m^−3^, organic carbon content of 0.23% m/m, and organic matter content of 14.17 g/kg); and Chengzi Experimental Station of Chifeng Academy of Agriculture Sciences, Chifeng (118.93°, 42.29°, sandy soil with a pH of 7.1, bulk density of 1,586 kg.m^−3^, organic carbon content of 0.21% m/m, and organic matter content of 13.63 g/kg). This constituted a 2-year, three-location trial, the pedigree information of 20 tested hybrids and the rainfalls of three locations are shown in Tables 1 and 2. Collection of plant material, must fully comply with relevant institutional, national, and international guidelines and legislation.

**Table 1:** Pedigree of 20 maize hybrids used in this study.

Code	Hybrid	Pedigree (♀ × ♂)	Origin	Active accumulated temperature for ripening (≥ 10 ℃)
G1	FT101	F1417 × T904	China	2750 ℃
G2	QL368	NK11 × NK17-8	China	2850 ℃
G3	JY5	J773 × J882	China	2850℃
G4	JA130	A626 × N215	China	2600 ℃
G5	LH5	LHM1620 × LA028	China	2600 ℃
G6	KD5112	KD9082 × KD9012	China	2800 ℃
G7	DH618	521 × DH392	China	2600 ℃
G8	NF99	NT218 × H581	China	2900 ℃
G9	XY335 (CK)	PH6WC × PH4CV	USA	2750 ℃
G10	LH1	M1001 × F2001	China	2800 ℃
G11	DK159	HCL301 × F0147Z	USA	2650 ℃
G12	XY1331	PH1CPS × PH26J9	USA	2600 ℃
G13	DF30	A311 × PH4CV	China	2600 ℃
G14	JKY3306	N16082 × X1267	China	2750 ℃
G15	JKY3308	F117 × A4190	China	2600 ℃
G16	HN887	B8-2-1 × Jing66	China	2900 ℃
G17	ZX7	M52 × M55	China	2500 ℃
G18	DM3307	R37 × P2	China	2600 ℃
G19	XM6	J203 × 817-2	China	2700 ℃
G20	CD228	C8-746 × HongC7-2	China	2900 ℃

**Table 2:** Mean monthly and total rainfall (mm) during the study period in 2018 and 2019 at three field sites.

Site	Year	Apr.	May	Jun.	Jul.	Aug.	Sep.	Oct.	Total
Chifeng	2018	20.1	11.3	77.9	119.0	136.8	26.2	3.2	394.5
	2019	14.5	93.0	46.5	65.2	98.7	15.5	22.9	356.1
Bayannur	2018	3.4	5.7	10.9	46.1	90.5	42.4	4.1	203.1
	2019	11.0	10.4	21.7	25.0	19.8	14.2	10.2	112.2
Salaqi	2018	27.3	61.0	11.8	189.1	108.4	84.7	9.9	492.1
	2019	29.6	19.3	70.6	85.6	100.3	72.4	26.2	404.0

### Experimental design

The experiment was conducted using a randomized complete block design. The 20 tested hybrids underwent two replications of drought-tolerant trials. The experimental plots were arranged in four rows, with a row spacing of 60 cm and a row length of 5 m. The planting density was determined to be 75,000 plants per hectare. Four protective rows were included around the experimental area. The plants were only watered before sowing, and no additional irrigation was provided throughout the entire growth period. During harvest, the two middle rows were selected, and the first and last plants from each row were removed for yield measurement.

### Statistical analysis

In this experiment, the AMMI model was employed to analyze the interaction between genotypes and environments, effectively capturing the interaction components of each genotype or environment. The AMMI model for analyzing yield data in maize hybrids is represented by the following equation^10,24^:

$$ {\text{Y}}_{{{\text{ge}}}} = \mu + \alpha_{{\text{g}}} + \beta_{{\text{e}}} + \, \lambda_{{\text{n}}} \gamma_{{{\text{gn}}}} \eta_{{{\text{en}}}} + \theta_{{{\text{ge}}}} , $$

where Y_ge_ represents the yield of genotype (G) in the environment (E); μ is thegrand mean; α_g_ is the genotype average deviation; λ_n_ is the eigenvalue of the n^th^principal component (PCA) axis, N is the total number of PCA, γ_gn_ and η_en_ are the genotype and environmental PCA scores for the n th PCA axis, and θ_ge_ is the residual.

The AMMI stability value (ASV) was calculated according to Purchase, Hatting and Van Deventer^18,25^ as follows:

$$\mathrm{ASV}=\sqrt{ [\frac{\mathrm{IPCA}1\mathrm{SS}}{\mathrm{IPCA}2\mathrm{SS}}}{(\mathrm{IPCA}1\mathrm{Score})]}^{2}+{[\mathrm{IPCA}2\mathrm{Score})]}^{2},$$

where SS is the sum of squares of the IPCAs and IPCA1 and IPCA2 are the first and second interaction principal component axes, respectively. Means of the genotypes were used for GGE biplot analysis.

GGE biplot: The grain yield data collected from three experimental sites were organized into a three-column data table of genotype-environment-yield, where each value represents the average yield of the corresponding genotype in the respective environment, known as the phenotype value (Yger). The linear statistical model for GGE biplot analysis is presented as follows^16,26^:

$$ {\text{Y}}_{{{\text{ger}}}} = \, \mu \, + \, \beta_{{\text{e}}} + \, \rho {\text{ge }} + \, \varepsilon {\text{ger }} + \, \sum \, \lambda {\text{n}}\gamma {\text{gn}}\delta {\text{ge}}, $$

where Y_ger_ represents the yield value of genotype g in environment e for the rth replication; μ is the overall mean; β_e_ represents the main effect of environment e; ρge is the residual of genotype g in environment e; εger represents the overall error; λn is the singular value of the nth principal component; γgn is the genotype g’s score for the nth eigenvector; δge is the environment e's score for the nth eigenvector. The parameters λnγgn and γnδen are defined as the GGE principal component scores for genotype g and environment e, respectively, also known as IPCAn or PCn. The data analysis was performed using Microsoft Excel 365 and Genstat 23 software on the Windows operating system.

## Results

### AMMI analysis of grain yield for drought-resilient maize hybrid selection

**Figure 1:**
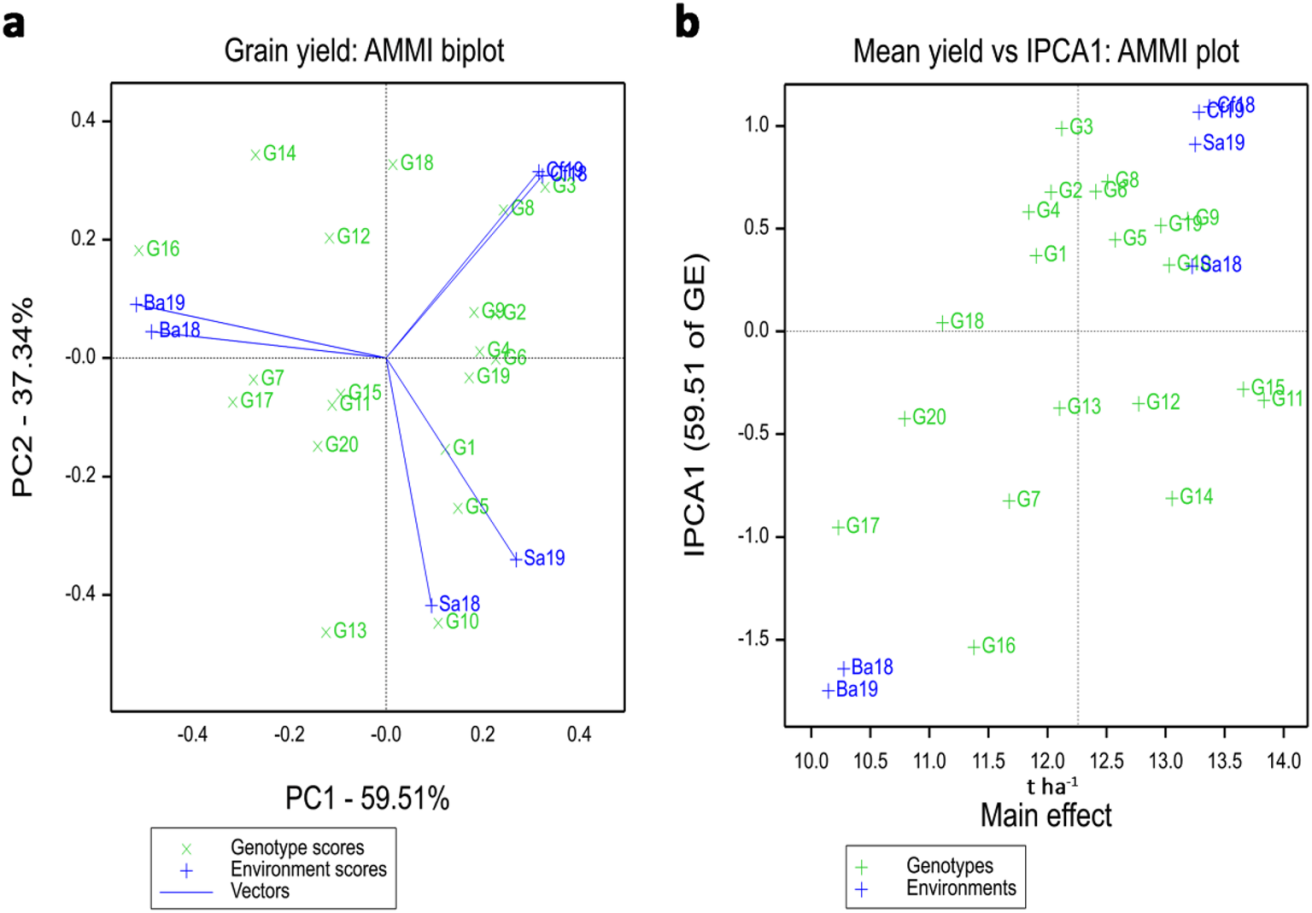
AMMI biplot showing relationship among test environments and genotypes based on grain yield. (**a**)Vector view of the AMMI biplot. The genotype scores were presented by green crosses and environments by blue pluses with vectors connecting the environment with the origin. Dotted vertical and horizontal lines indicate points where the PC1 and PC2 axes had respective values of zero. See codes of genotypes (G1 to G20) in Table 3. Ba18, Bayannur 2018; Ba19, Bayannur 2019; Sa18, Salaqi 2018; Sa19, Salaqi 2019; Cf18, Chifeng 2018; Cf19, Chifeng 2019. (**b**) AMMI1 biplot for additive effects vs IPCA1. The genotype scores were presented by green crosses and environments by blue pluses. Dotted vertical and horizontal lines indicate points where the IPCA1 and mean grain yield axes had respective values of zero.

The average grain yield differences of the experimental hybrids varied widely, ranging from 8.46 to 15.94 ton per hectare. Table 3 displays the two-year grain yield of the 20 maize hybrids across three environments in Inner Mongolia. AMMI variance analysis revealed highly significant (P < 0.001) main effects for genotype, environment, and their interaction. The interaction between genotypes and environments were decomposed into interaction principal component axes 1 (IPCA1), interaction principal component axes 2 (IPCA2) and interaction principal component axes 3 (IPCA3) (Table 4). IPCA1 and IPCA2 were found to be highly significant, explaining 59.51% and 37.34% of the total variation of G × E interaction (Fig. 1a), respectively. G11 (DK159) exhibited the highest average yield across all tested sites (Fig. 1b and Table 3) and also demonstrated broad adaptability, as indicated by its proximity to the origin (Fig. 1a). In contrast, G16 and G13 were highly influenced by environmental interaction (Fig. 1a). All of the environments were positioned far from the origin, indicating strong interaction forces with genotype, and the angles between the tested environments suggested distinctiveness in selecting drought-tolerant hybrids (Fig. 1a).

**Table 3:** Mean grain yield (t ha^–1^) of 20 maize hybrids in three environments under rain-fed condition in Inner Mongolia.

Code	Bayannur		Salaqi		Chifeng		Yield t ha^–1^
	2018	2019	2018	2019	2018	2019	
G1	9.02	8.73	13.87	13.55	12.93	12.76	11.81
G2	8.99	8.76	12.98	13.30	13.01	13.34	11.73
G3	8.83	8.46	11.72	13.47	13.23	12.81	11.42
G4	8.73	8.95	12.71	13.59	14.42	14.18	12.10
G5	10.29	8.92	14.78	14.78	13.64	13.44	12.64
G6	9.06	9.36	13.47	14.18	11.40	12.04	11.59
G7	10.66	11.33	12.45	12.17	14.32	14.05	12.50
G8	9.30	9.40	13.04	12.90	14.07	14.10	12.14
G9	10.34	10.16	14.16	14.24	12.93	12.89	12.45
G10	10.48	9.81	15.91	15.94	13.90	14.25	13.38
G11	12.14	12.42	15.19	14.65	15.30	14.93	14.10
G12	11.48	11.44	12.75	12.76	11.33	11.30	11.84
G13	10.58	10.17	14.89	14.38	14.34	14.28	13.11
G14	12.73	12.55	12.12	12.31	14.45	13.99	13.02
G15	12.08	12.02	14.76	14.64	14.99	15.42	13.99
G16	9.93	9.50	10.93	10.25	10.05	9.51	10.03
G17	12.17	11.99	11.55	10.49	11.53	11.16	11.48
G18	9.30	9.08	11.01	10.68	13.42	13.18	11.11
G19	10.21	9.83	14.17	14.59	14.56	14.38	12.96
G20	9.18	9.56	12.08	12.12	10.96	10.84	10.79
Mean	10.28	10.12	13.23	13.25	13.24	13.14	12.21
Min	8.73	8.46	10.93	10.25	10.05	9.51	10.03
Max	12.73	12.55	15.91	15.94	15.30	15.42	14.10
Std	1.27	1.33	1.45	1.55	1.47	1.51	1.04
CV (%)	0.12	0.13	0.11	0.12	0.11	0.12	0.09

**Table 4:** Variance analysis by AMMI model.

Source	d.f	s.s	m.s	v.r	F pr
Total	239	4,551,024	19,042		
Treatments	119	4,243,600	35,661	19.51	< 0.001
Genotypes	19	1,040,202	54,747	29.95	< 0.001
Environments	5	2,159,025	431,805	26.17	< 0.001
Block	6	99,016	16,503	9.03	< 0.001
Interactions	95	1,044,373	10,993	6.01	< 0.001
IPCA 1	23	702,416	30,540	16.71	< 0.001
IPCA 2	21	294,837	14,040	7.68	< 0.001
IPCA 3	19	28,472	1499	0.82	0.6799
Residuals	32	18,648	583	0.32	0.9998
Error	114	208,408	1828

### GGE biplot analysis of G × E interaction

#### Which won where model

**Figure 2:**
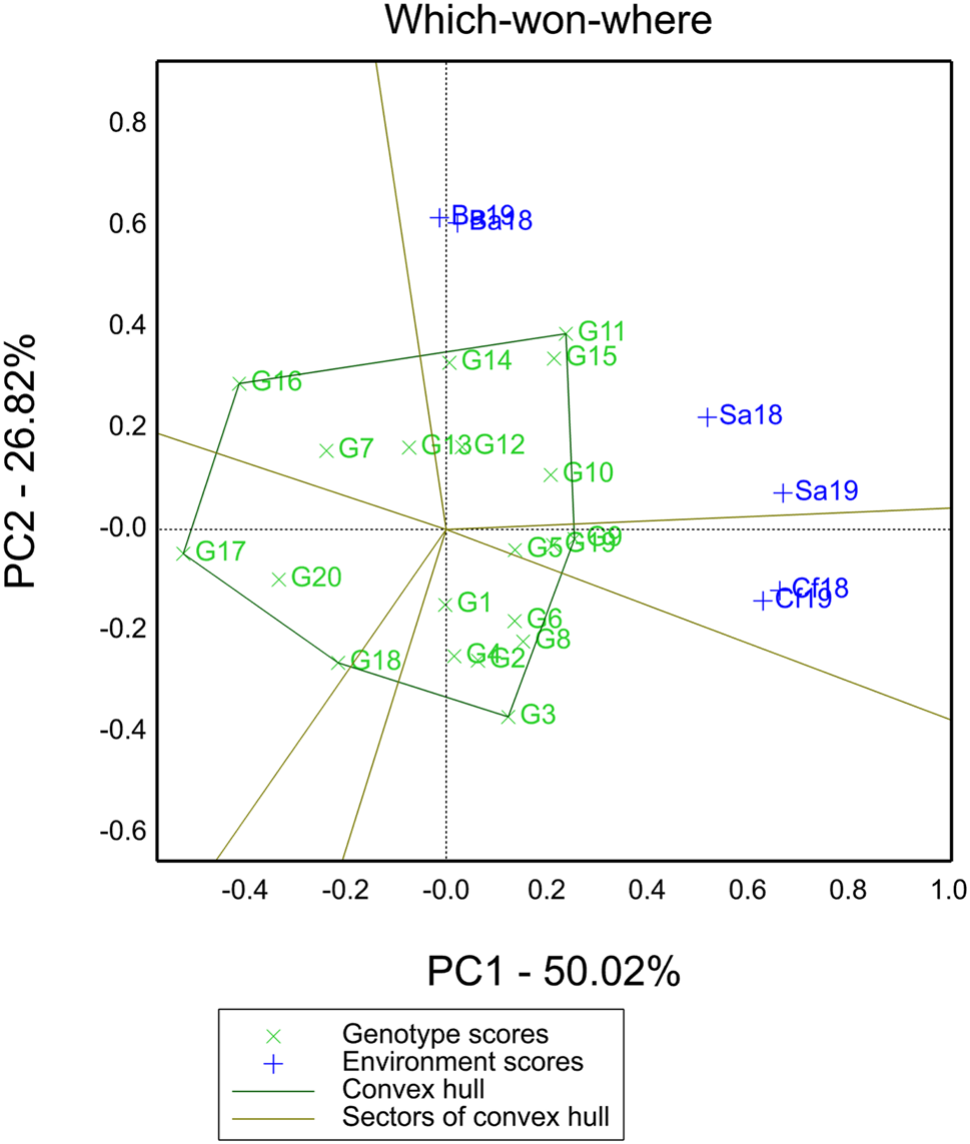
The “which-won-where” view of the GGE biplot showing which genotypes performed best in which environment. Dotted vertical and horizontal lines indicate points where the PC1 and PC2 axes had respective values of zero. Vertices of the polygon indicate superior genotypes in each sector. See the code of genotype in Table 3 and tested sites abbreviations in Fig. 1, the same below.

A “which-won-where” polygon view was presented, illustrating the relationship between genotypes and environments (Fig. 2). The biplot analysis accounted for 76.84% of the total observed variation, with 50.02% explained by the first principal component (PC1), and 26.82% by the second principal component (PC2). Genotypes G11, G9, G3, G18, G17 and G16 were situated at the corners of the “which-won-where” polygon, indicating their exceptional performance in specific environments (Yan and Ticker. 2006). Among these genotypes, G11 exhibited the highest grain yield in four out of six tested environments: Ba18, Ba19, Sa18, and Sa19. The CK (G9) outperformed in terms of yield in the Cf18 and Cf19 environments(Fig. 2).

### Environmental vector view

**Figure 3:**
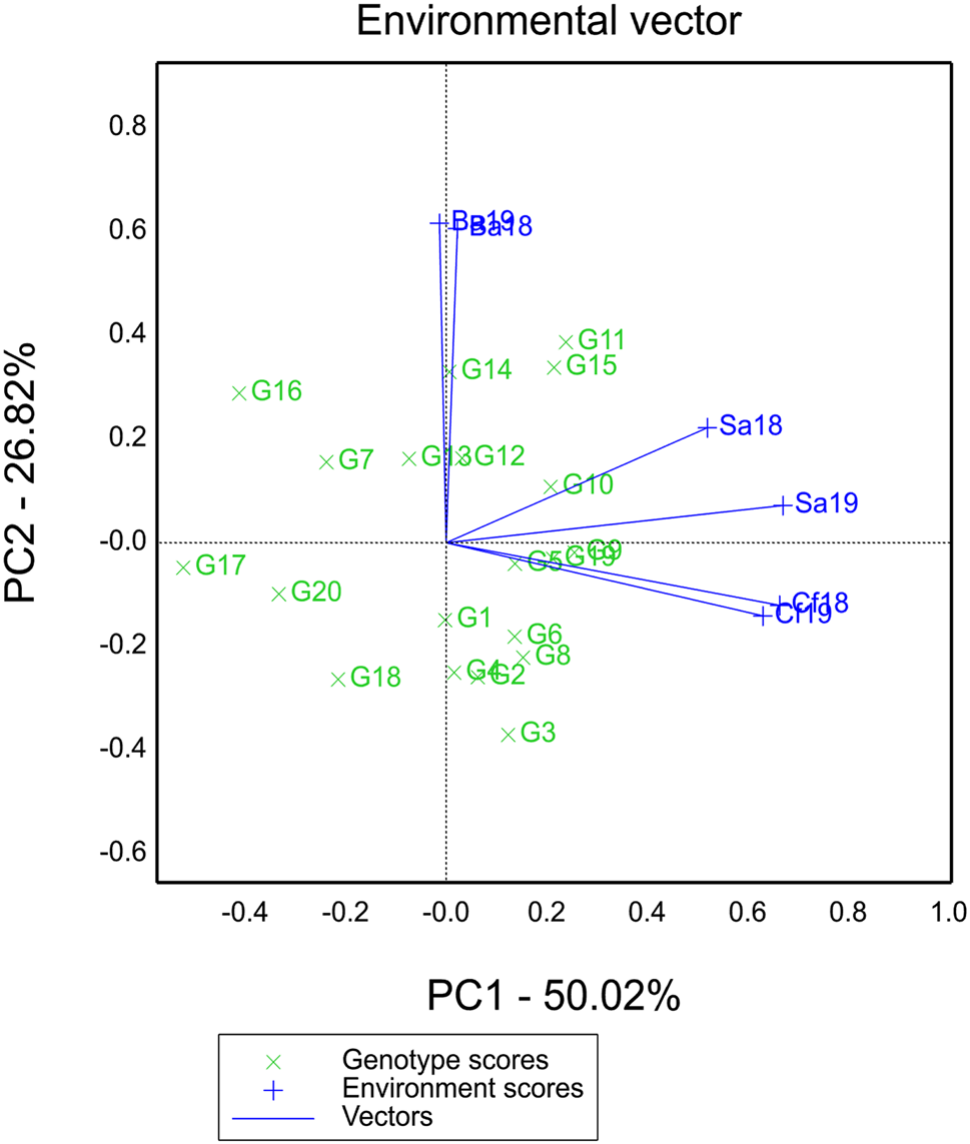
Environmental vector view of GGE biplot.

The "Environmental vector view" function plot of GGE biplot was utilized to analyze the hybrids. The angles between the Ba and Cf environments were greater than 90 degrees, suggesting a negative correlation and opposite ranking of hybrids between these two environments (Fig. 3). On the other hand, the angles between all other environments were less than 90 degrees, indicating a positive correlation among them. The length of the environmental line represents the discrimination ability of the test sites for the hybrids. Among the test environments, Ba18 had the strongest the discrimination ability (Table S1). The mega-environment function plot revealed that Cf formed a distinct type, while Ba and Sa belonged to another type (Fig. 2 and Supplemental Fig. 1).

### Ranking biplot mean vs stability view

**Figure 4:**
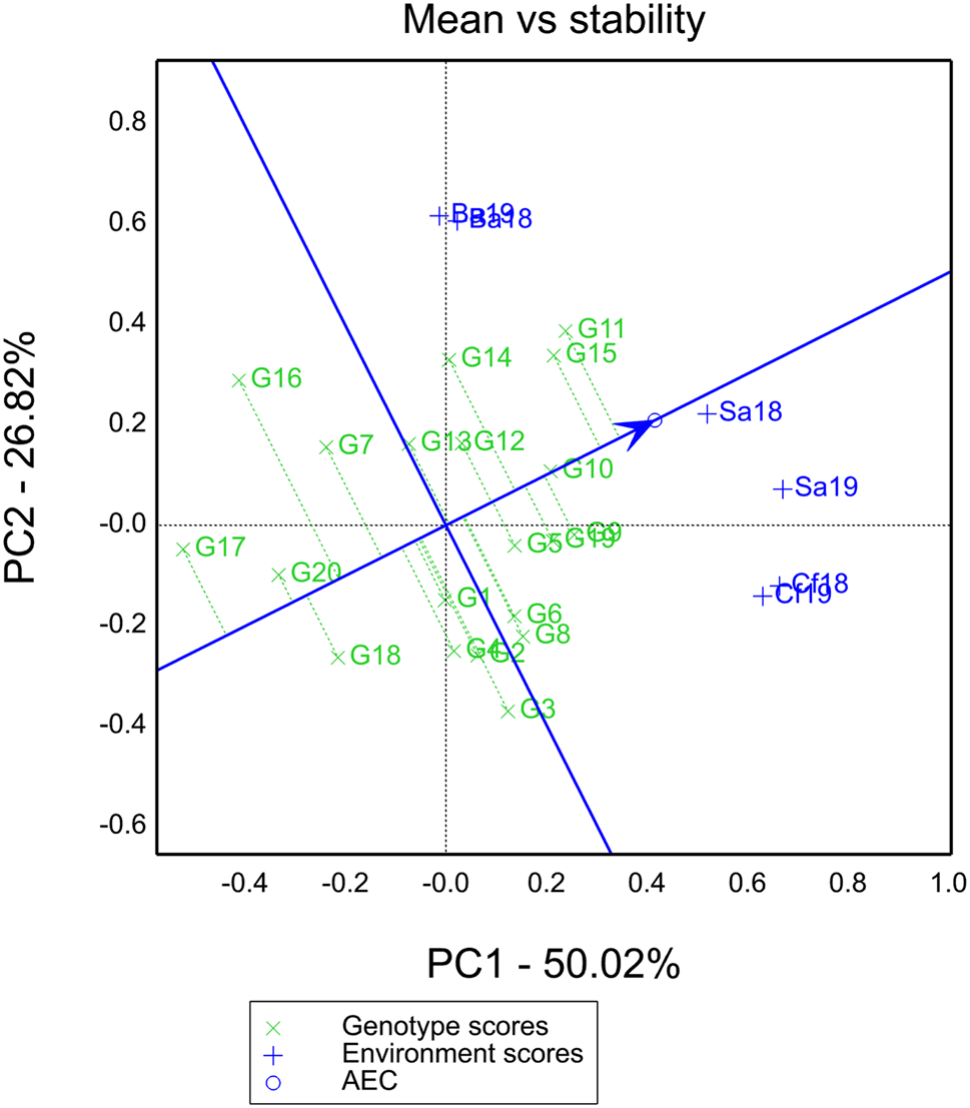
Mean vs stability view of ranking biplot. The straight line with arrows is the environmental average axis. The perpendicular length from the average axis to the genotypes indicates the stability of each genotype. A longer perpendicular length signifies a higher level of instability for that genotype.

In the positive direction, the biplot indicates that G11 has the closest projection onto the Average Environment Coordinate (AEC) axis, suggesting G11 exhibited significantly higher yield compared to the other hybrids. The stability analysis of each hybrid shows that G10 have the shortest perpendicular distance to the AEC axis, indicating the highest stability in yield. Conversely, G16 and G3 have the longest vertical distance, indicating the lowest stability in grain yield (Fig. 4).

### Best hybrid and best environment by GGE biplot

**Figure 5:**
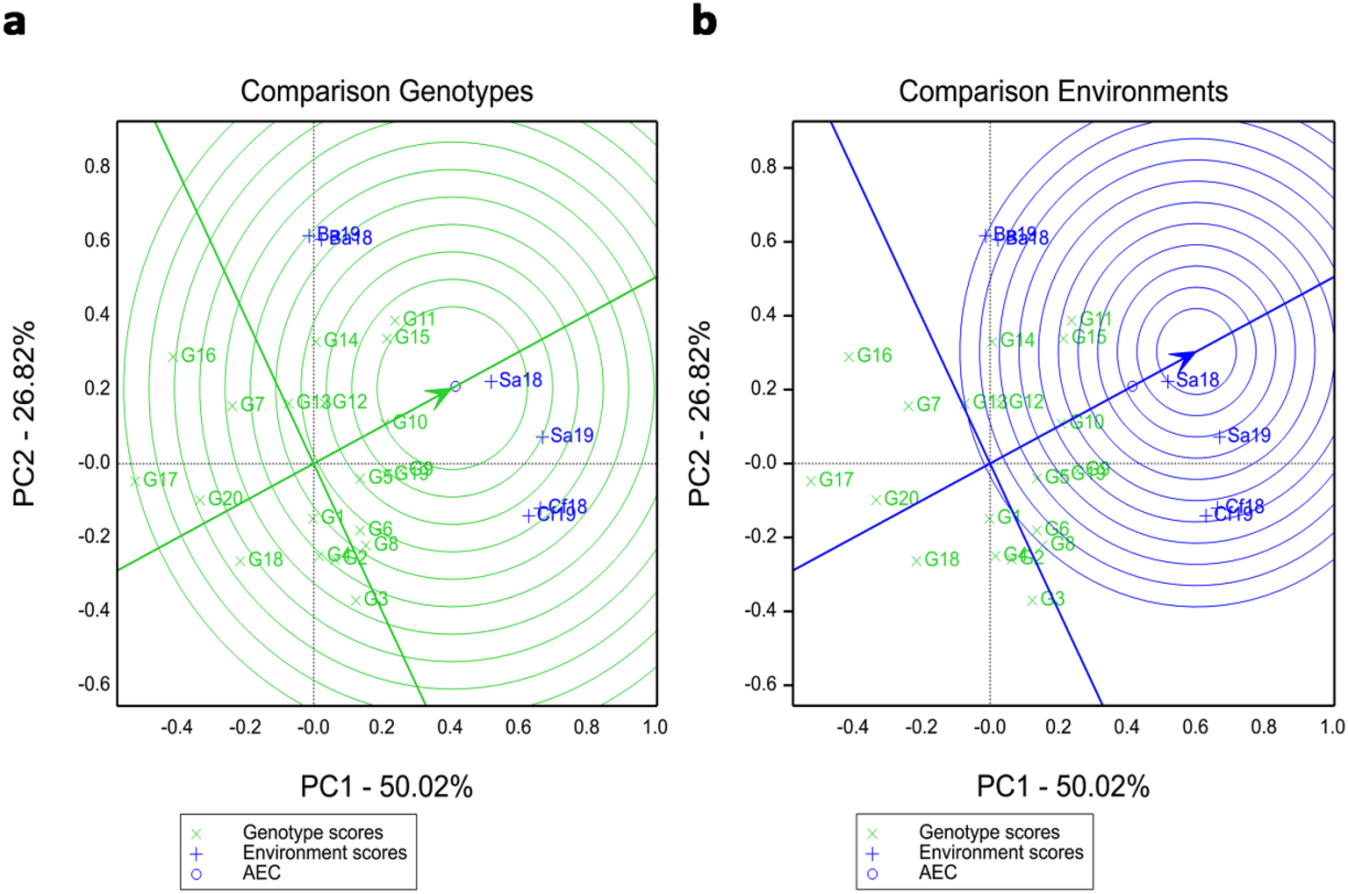
Best hybrid and best environment by GGE biplot. (**a**) Best hybrid view of GGE biplot comparing hybrids relative to an ideal hybrid. (**b**) Best environment view of GGE biplot comparing environments relative to an ideal environment.

Based on the best genotype comparison biplot, the top-performing hybrid across the three tested environments was G10, followed by G15 and G11, which consistently displayed above-average grain yield in all environments (Fig. 5a). Other desirable genotypes, including G9 and G19, which were located on the second concentric circles, respectively. The AEC view comparing environments relative to an ideal environment is presents. It indicates that environments Sa18 and Sa19 were closer to the center of the concentric circle (Fig. 5b). Compared to the other two locations, Salaqi was identified as the ideal environment (Fig. 5b).

## Discussion

The significant G × E effects observed in recent study indicate that the evaluated genotypes do not exhibit consistent performance across different test environments^27–30^. This highlights the importance of investigating the nature and magnitude of G × E, which cannot be adequately captured by a standard analysis of variance^18,31^. The AMMI model, which combines PCA and analysis of variance, allows for a comprehensive analysis of genotype and environment interactions and facilitates the identification of interaction patterns^32^. IPCA1 of our AMMI analysis contributed 59.51% to the total variation across the tested environments, which implies genotypes and environments have strong interaction^33^. IPCA1 and IPCA2 accounted for 96.85% of the interaction sum of squares. As a result, IPCA3 did not achieve significantly difference (Table 4). AMMI with the first two multiplicative terms was deemed to be the best predictive model in a previous study^24^. On the other hand, the GGE biplot visually presents data in a graph form, providing an intuitive visualization of the specific characteristics of varieties^7,27^. It complements the AMMI model by offering a graphical representation that facilitates the interpretation of G × E interactions^28,30^.

The AMMI stability values, such as the ASI and ASV, provide additional information on the variation among genotypes^20^. The genotypes with ASV values close to zero are considered stable^34^. In our study, G15 (JKY3308) exhibited an ASV of 3.906, suggesting it may possess genes for adaptability to various agroclimatic conditions (Table S2). However, it is important to note that while G15 ranked as one of the top stable hybrids based on the GGE biplot (Fig. 5), it may not be the most stable hybrid. Therefore, there might be some differences in the results obtained from the AMMI and GGE biplot analyses. To obtain a more reliable analysis, breeders often combine the insights gained from both approaches^7,35,36^.

The selection of appropriate test locations plays a crucial role in crop breeding programs^37,38^. The effectiveness and accuracy of variety selection are directly influenced by the identification ability of the test environments^39^. In this study, we focused on evaluating three rain-fed regions in Inner Mongolia, each characterized by distinct geographical and ecological conditions, using the AMMI model and GGE biplot (Fig. 1a). An environment with long vector and limited angle offers a richer and more accurate representation^38^. Our findings revealed that the test site in Bayannur demonstrated a stronger overall identification ability throughout the two-year period compared to the other two environments (Fig. 3, Tables 2 and S1). This can be attributed to factors such as lower average annual rainfall and significant day-night temperature differences in Bayannur. In future studies, we intend to incorporate additional ecological test environments and rainproof chambers to further enhance our drought tolerant hybrids identification programs. This will allow us to accurately evaluate the stability and adaptability of hybrids or elite inbred lines. By expanding the scope of our evaluations, we aim to improve the precision and reliability of hybrid selection in maize breeding.

## Conclusion

Our comprehensive analysis has revealed insights into the performance of maize in the drought-tolerant hybrid selection trials by AMMI model and GGE biplot. AMMI variance analysis revealed highly significant difference on main effects for genotype and environment. G11 (DK159) and G15 (JKY3308) exhibited highest productivity and stability among 20 hybrids. G10 (LH1) was identified as the most stable hybrid across the three test environments. Three test sites belong to distinctive rain-fed types and Bayannur test site exhibited the highest identification ability among them. The integration of the AMMI model and GGE biplot has provided a robust and comprehensive approach for evaluating and identification of drought-resilient maize hybrids in drought-prone regions of China or similar environments.

### Supplementary Information

Supplementary Information.

## Data Availability

All data generated or analyzed during this study are included in this published article and its supplementary information files.
