# Supplementary material for: AMMI an GGE biplot analysis of grain yield for drought-tolerant maize hybrid selection in Inner Mongolia
Source: Sci Rep. 2023 Nov 1;13:18800. doi: 10.1038/s41598-023-46167-z (PMC10620424; doi:10.1038/s41598-023-46167-z)
Supplement: Supplementary file 1 — Supplementary Information. [file 41598_2023_46167_MOESM1_ESM.docx]

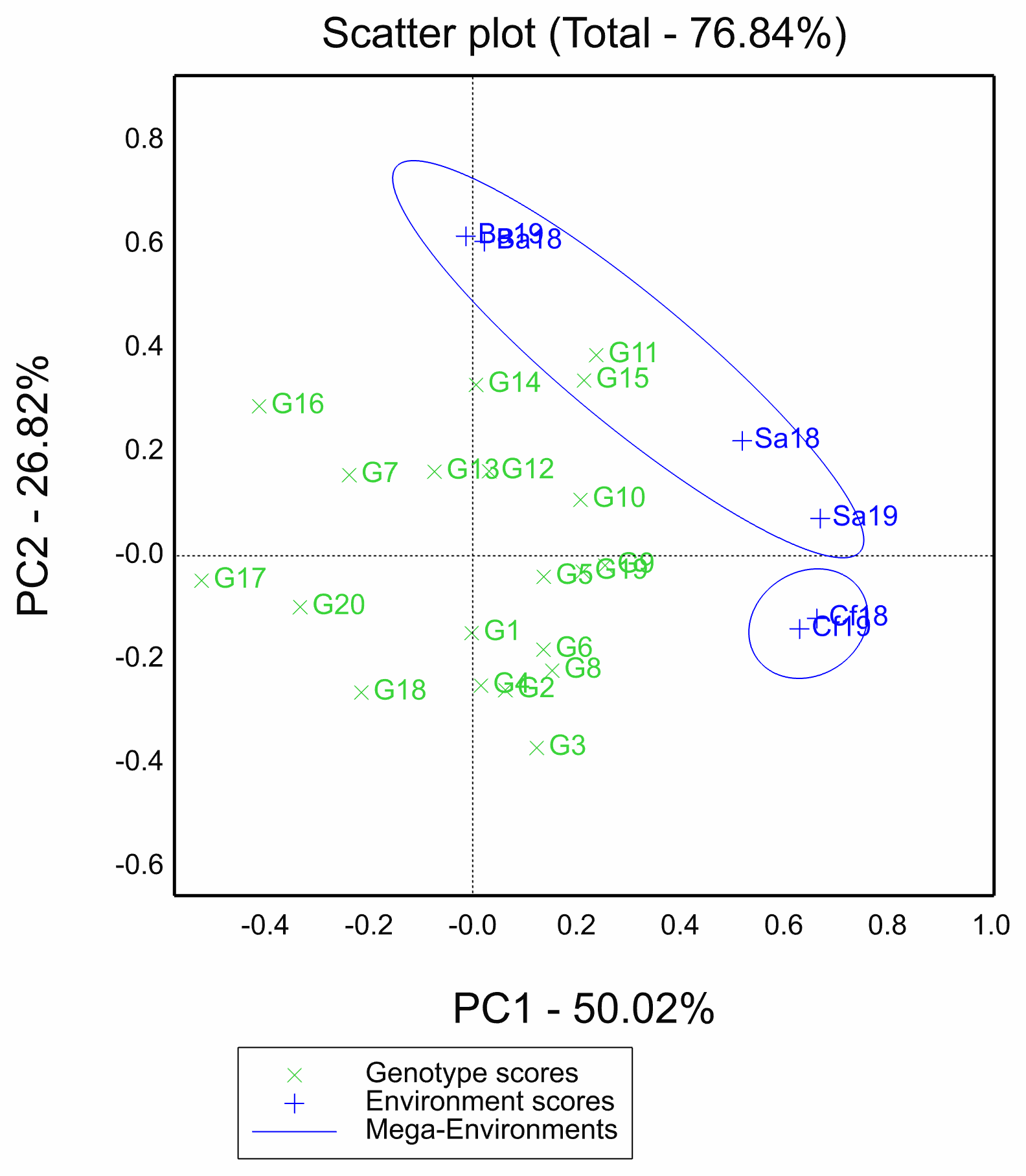


Fig. S1 the Scatter plot of environment types. See the code of genotype in Table 1 and tested sites abbreviations in Figure 1.

Table S1 Environment means and scores of AMMI model

Environment Number Mean IPCAe1 IPCAe2

Ba18 1 685.0 -13.39776 -1.37804

Ba19 2 676.4 -14.27682 -2.80525

Cf18 3 891.5 8.93031 -9.56149

Cf19 4 885.5 8.71758 -9.77350

Sa18 5 881.7 2.59181 12.96283

Sa19 6 883.4 7.43487 10.55545

Table S2 AMMI stability values of the 20 tested hybrids.

| Rank | Code | Number | Stability (ASV) | Mean |
| --- | --- | --- | --- | --- |
| 1 | G15 | 7 | 3.906 | 13.66 |
| 2 | G11 | 3 | 4.710 | 13.83 |
| 3 | G1 | 1 | 5.855 | 11.90 |
| 4 | G12 | 4 | 6.372 | 12.77 |
| 5 | G20 | 13 | 6.422 | 10.79 |
| 6 | G19 | 11 | 6.749 | 12.96 |
| 7 | G18 | 10 | 7.157 | 11.11 |
| 8 | G9 | 20 | 7.298 | 13.19 |
| 9 | G4 | 15 | 7.568 | 11.84 |
| 10 | G5 | 16 | 8.013 | 12.57 |
| 11 | G6 | 17 | 8.858 | 12.41 |
| 12 | G2 | 12 | 8.963 | 12.03 |
| 13 | G10 | 2 | 10.633 | 13.03 |
| 14 | G7 | 18 | 10.757 | 11.68 |
| 15 | G8 | 19 | 10.943 | 12.51 |
| 16 | G13 | 5 | 11.220 | 12.10 |
| 17 | G17 | 9 | 12.504 | 10.23 |
| 18 | G14 | 6 | 12.944 | 13.05 |
| 19 | G3 | 14 | 14.310 | 12.12 |
| 20 | G16 | 8 | 20.380 | 11.38 |
